# Gene therapy for inherited retinal diseases: exploiting new tools in genome editing and nanotechnology

**DOI:** 10.3389/fopht.2023.1270561

**Published:** 2023-09-19

**Authors:** Cláudia Carvalho, Luísa Lemos, Pedro Antas, Miguel C. Seabra

**Affiliations:** ^1^ iNOVA4Health, NOVA Medical School | Faculdade de Ciências Médicas, NMS | FCM, Universidade Nova de Lisboa, Lisboa, Portugal; ^2^ Champalimaud Research, Champalimaud Foundation, Lisboa, Portugal

**Keywords:** inherited retinal diseases, gene therapy, genome editing, CRISPR-Cas, nanoparticles

## Abstract

Inherited retinal diseases (IRDs) encompass a diverse group of genetic disorders that lead to progressive visual impairment and blindness. Over the years, considerable strides have been made in understanding the underlying molecular mechanisms of IRDs, laying the foundation for novel therapeutic interventions. Gene therapy has emerged as a compelling approach for treating IRDs, with notable advancements achieved through targeted gene augmentation. However, several setbacks and limitations persist, hindering the widespread clinical success of gene therapy for IRDs. One promising avenue of research is the development of new genome editing tools. Cutting-edge technologies such as CRISPR-Cas9 nucleases, base editing and prime editing provide unprecedented precision and efficiency in targeted gene manipulation, offering the potential to overcome existing challenges in gene therapy for IRDs. Furthermore, traditional gene therapy encounters a significant challenge due to immune responses to viral vectors, which remain crucial obstacles in achieving long-lasting therapeutic effects. Nanotechnology has emerged as a valuable ally in the quest to optimize gene therapy outcomes for ocular diseases. Nanoparticles engineered with nanoscale precision offer improved gene delivery to specific retinal cells, allowing for enhanced targeting and reduced immunogenicity. In this review, we discuss recent advancements in gene therapy for IRDs and explore the setbacks that have been encountered in clinical trials. We highlight the technological advances in genome editing for the treatment of IRDs and how integrating nanotechnology into gene delivery strategies could enhance the safety and efficacy of gene therapy, ultimately offering hope for patients with IRDs and potentially paving the way for similar advancements in other ocular disorders.

## Introduction

Inherited retinal diseases (IRDs) are a group of genetic disorders that cause progressive retinal degeneration, leading to severe visual impairment or complete vision loss. IRDs have an estimated global prevalence of approximately 1 in 2000 individuals (1), representing the leading cause of blindness among the working-age population in the Western world (2). Most of these blinding conditions stem from monogenic mutations primarily expressed in the retinal pigment epithelium (RPE) and/or photoreceptors, crucial for supporting the retina and converting light into electrical signals. The last decades have witnessed tremendous progress towards unraveling the genetic basis of IRDs. Nearly 300 causative genes have been identified, each often harboring multiple disease-causing variants with distinct clinical phenotypes (https://sph.uth.edu/retnet/). This vast genetic heterogeneity accounts for highly heterogeneous clinical presentations, with variable symptoms, inheritance mode, onset age, progression rate, and severity. Among the most common IRD subtypes are Leber congenital amaurosis (LCA), Retinitis Pigmentosa (RP), and Choroideremia.

For a long time, IRDs were deemed largely incurable diseases. However, the landscape of treatment options is now rapidly evolving due to remarkable advances in molecular genetic testing and gene therapy development. Over the last few decades, gene therapy has emerged as a compelling approach owing to the monogenic nature of most IRDs, their well-established genetic etiology, and the unique advantageous features of the eye, such as easy physical access and immune-privileged status (3). Among the existing gene therapy modalities, gene supplementation through adeno-associated viral (AAV) vectors is the most widely explored and is rapidly gaining ground in the clinic. Almost a decade has passed since we published the results of the first phase I/II gene therapy clinical trial for Choroideremia (NCT01461213) (4, 5), which evaluated the safety and efficacy of AAV-REP1 supplementation therapy. This clinical trial met its primary endpoint of improving vision in treated eyes, with visual acuity gains sustained for up to 5 years. Despite the initial promise, the therapy was discontinued after Biogen’s Phase III multicenter randomized clinical trial (NCT03496012) failed to meet primary and secondary endpoints. Parallel research efforts have been dedicated to the development of AAV-mediated supplementation therapy targeting biallelic *RPE65* mutations. Encouraging early results from the phase III trial (6) have culminated in the regulatory approval of Luxturna (voretigene neparvovec) in 2017, bringing to light the first and only gene therapy for an IRD to date.

With the clinical success of Luxturna, gene supplementation has risen to the forefront of ocular gene therapy research. However, this treatment modality is only suited for IRDs caused by recessive mutations, leaving dominant IRDs beyond its scope. Unlike recessive diseases, dominant conditions typically result from gain-of-function or dominant-negative mutations that confer a new pathogenic role to the encoded protein. Therefore, to treat dominant IRDs, it does not suffice to supplement the faulty gene with a healthy copy; instead, the therapy must also inhibit the expression of the toxic gene product to mitigate its harmful effects. Adding to its restricted applicability, the durability of therapeutic effects achieved through gene supplementation remains controversial. While some long-term clinical studies of Luxturna show that visual function gains are sustained up to 4 and 7.5 years (7–9), other *RPE65* gene therapy trials report unabated retinal degeneration and relapse in visual acuity a few years after treatment (10–12). Collectively, these limitations underscore the urge to explore alternative strategies to gene supplementation for addressing the broad spectrum of IRDs.

CRISPR-based genome editing has been attracting widespread interest as a potential solution to the shortcomings of gene supplementation. CRISPR is an acronym for “clustered, regularly interspaced, short palindromic repeats” and refers to a programmable tool that enables highly precise modification, removal, and replacement of target DNA sequences. By permanently correcting the underlying mutation, this technology halts the expression of the mutant protein, eliminates the concerns for declining transgene expression over time, and allows for physiologically regulated expression of the corrected gene. As a result, CRISPR-based genome editing has the potential to translate into sustained therapeutic effects and extend the therapeutic application to dominant diseases, holding promising implications for the future of IRD treatment.

This article provides a comprehensive overview of the latest advancements in diverse CRISPR-based genome editing techniques for IRDs. While acknowledging the progress achieved, we also address the remaining challenges, emphasizing the pressing need for alternative approaches surpassing AAV-mediated gene delivery’s limitations. By exploring these novel avenues, we aim to unlock new possibilities for the effective treatment of IRDs and pave the way for transformative solutions in the field of ocular disorders.

## CRISPR/Cas nuclease editing in the context of IRDs

**Figure 1:**
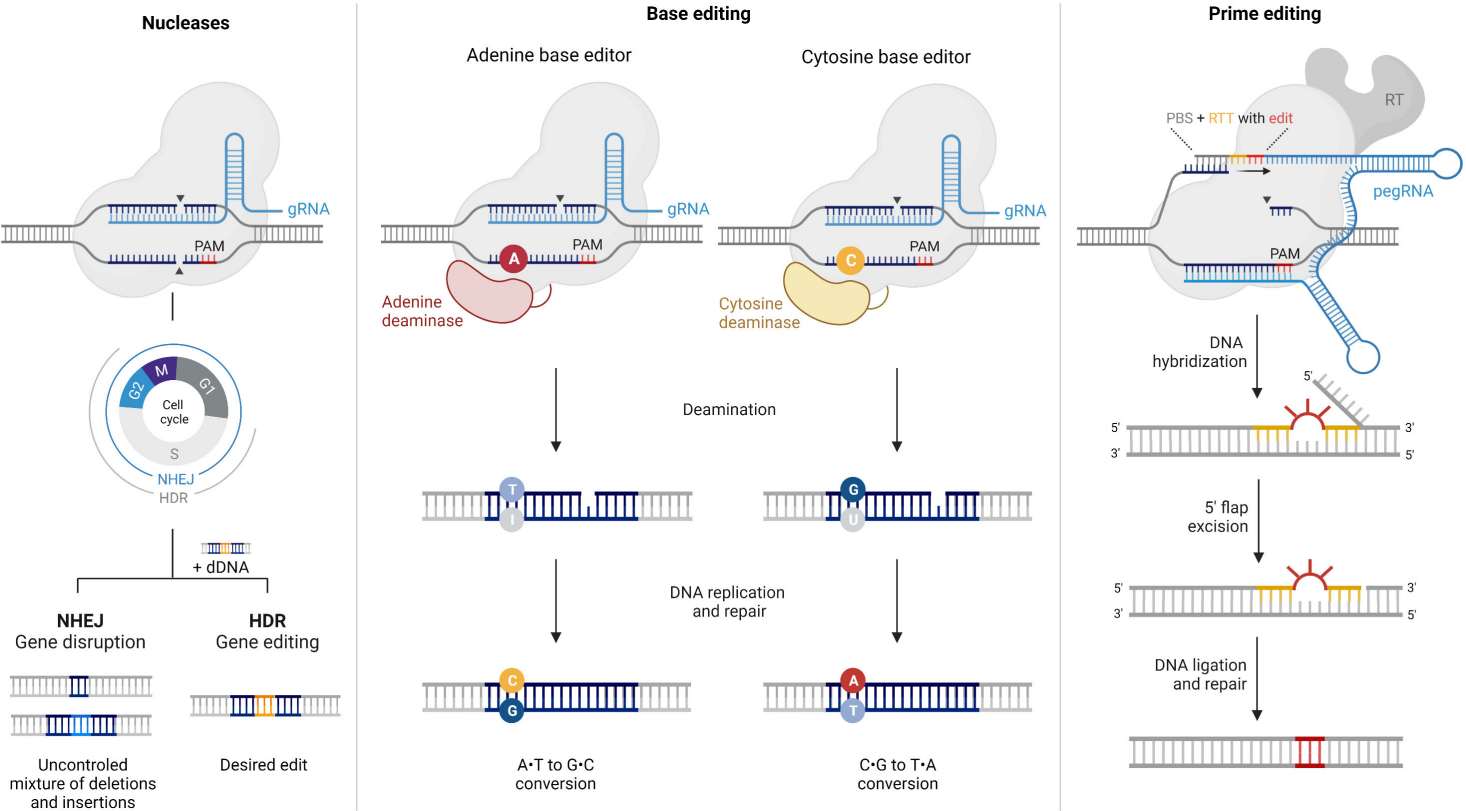
Comparison of CRISPR/Cas9 nucleases, base editors, and prime editors. This figure provides an overview of the components and mechanisms of action of CRISPR/Cas9 nucleases, base editors, and prime editors, highlighting their unique abilities in achieving specific genetic modifications with varying levels of precision. CRISPR/Cas9 is a powerful genome editing tool that uses a guide RNA (gRNA) to target specific DNA sequences within the genome. The Cas9 enzyme acts as molecular scissors, creating double-strand breaks (DSBs) at the targeted site. This prompts the cell’s natural repair machinery to introduce insertions or deletions by non-homologous end-joining (NHEJ), resulting in gene disruption or knockout. In the presence of a donor DNA (dDNA) template, DSBs can alternatively be corrected by homology-directed repair (HDR), provided that the cell is in the G2 or S phases of the cell cycle. Base editors are genome editing tools designed to introduce precise single-nucleotide mutations without causing DSBs. They utilize a modified Cas9 enzyme fused to a deaminase enzyme to convert a targeted DNA base pair (e.g., A•T to I•T) without disrupting the DNA backbone. Subsequently, the cell’s repair machinery converts the modified base into a desired substitution. Prime editing is a gene editing technique that allows the precise insertion, deletion, or substitution of DNA sequences at target sites, without requiring DSBs. Prime editors consist of a catalytically impaired Cas9 fused to an engineered reverse transcriptase and a prime editing guide RNA (pegRNA). The pegRNA directs the reverse transcriptase (RT) to introduce the desired edito into the target DNA. PBS: primer binding site, RTT: reverse transcription template.

CRISPR/Cas systems are revolutionizing precision medicine and hold immense promise for treating IRDs. The programmable CRISPR/Cas genome editing tool was initially derived from a bacterial adaptive immune system (13) and consists of a Cas protein loaded with a short RNA sequence named guide RNA (gRNA), specifically engineered to target the gene of interest (**Figure 1**). Once inside the cell, the Cas endonuclease stochastically searches for target DNA by binding to sequences that match its protospacer adjacent motif (PAM) sequence. When a region with the appropriate PAM and complementary to the gRNA is found, the Cas protein induces a site-specific double-strand break (DSB) that is promptly repaired by either non-homologous end joining (NHEJ) or homology-directed repair (HDR). The main difference between these two mechanisms is that NHEJ is an error-prone pathway that randomly repairs the DSB, generating insertions and deletions (indels) at the target site. In contrast, HDR uses a donor DNA template to install a desired sequence at a specific site, resulting in a more precise product (14).

The NHEJ pathway allows the disruption of coding sequences or regulatory motifs by excision, inversion, or frameshift mutations. Depending on the strategy adopted, this mechanism can be harnessed to address dominant and recessive conditions. When haplosufficiency mechanisms are present, autosomal dominant diseases can be ameliorated by selectively inactivating the mutant allele to mitigate its toxic effect and relying on the healthy allele to express the native protein. This strategy has proven successful in ablating the mutant *Rho* gene in rodent autosomal dominant RP (adRP) models, leading to delayed photoreceptor cell degeneration and improved retinal function (15–18). Alternatively, an approach suited for autosomal dominant and recessive diseases is to disrupt both alleles in a mutation-independent manner and then restore wild-type protein expression by gene supplementation (19–21). Ablate-and-replace gene therapies were first described by the research group led by Stephen Tsang, demonstrating ameliorated disease progression in two types of human *RHO* mutation knock-in mouse models of adRP (19). In addition, NHEJ can be utilized to correct splicing errors, ensuring proper pre-mRNA processing. This approach is at the basis of EDIT-101, an experimental CRISPR/Cas therapeutic developed for LCA type 10. EDIT-101 utilizes a pair of gRNAs to target and remove the aberrant splice donor created by the IVS26 mutation in *CEP290* gene, thereby restoring normal CEP290 protein. Subretinal delivery of EDIT-101 to mice and non-human primates was well tolerated and achieved sustained productive editing rates at levels that met or exceeded the target therapeutic threshold (22). These promising preclinical results culminated in the FDA-approval of the first clinical trial for in-body CRISPR gene therapy in September 2019 (NCT03872479). In November 2022, Editas Medicine provided a clinical update that identified homozygous patients as the responder population and demonstrated a favorable safety profile across all dose cohorts, with no reported drug-related serious adverse events (23). However, given the limited number of predicted responders, the pharmaceutical company paused enrollment in the trial and is now seeking to identify a collaboration partner to continue developing EDIT-101. Another interesting concept that has been proposed is to employ NHEJ-mediated genome editing to reprogram affected cell populations into functionally related cell types that are resistant to disease-causing mutations (24, 25). Zhu et al. established a dual AAV-CRISPR/Cas9 system to reprogram rods into cone-like photoreceptors by inactivating *Nrl* (rod fate determinant) or *Nr2e3* (*Nrl* downstream transcription factor) (25). The treatment significantly rescued rod and cone degeneration and restored visual function in two mouse autosomal recessive RP (arRP) models.

Although NHEJ is making considerable progress, HDR-mediated genome editing is typically considered more desirable for IRD treatment. This repair pathway offers greater control over the editing outcome and enables the correction of a broader spectrum of mutations. However, because HDR activity is restricted to the G2 and S phases of the cell cycle, it is highly inefficient in post-mitotic retinal cells and frequently outcompeted by concurrent NHEJ repair (26–29). This leads to high rates of bystander indel mutations, ultimately undermining the potential advantages of mutation repair by HDR. In an effort to enhance HDR activity in terminally differentiated retinal cells, Cai et al. incorporated bacterial recombinase A (RecA) into the CRISPR/Cas9 system to catalyze DNA exchange reactions (30). To evaluate HDR efficiency, the devised Cas9/RecA system was used to target the *Pde6b* nonsense mutation in postnatal *rd1* mice, a mutant model of arRP. Compared to unmodified Cas9, Cas9/RecA treatment precisely repaired *Pde6b* point mutation with enhanced HDR efficiency, significantly rescued photoreceptor degeneration, and partially restored the response to light stimulus. Nevertheless, the levels of functional rescue achieved were still far below those observed in wild-type mice and insufficient to guarantee clinical efficacy. Compounding this problem, an increasing number of reports have disclosed several adverse consequences associated with the generation of DSBs in genomic DNA, including large deletions (31, 32), chromosomal rearrangements (33, 34), chromothripsis (35), viral integration (36), and activation of p53 (37, 38). These safety risks, along with the inefficiency of HDR in most cell types, have encouraged the development of DSB-independent CRISPR/Cas systems, such as base editing and prime editing methods.

## Base editing propelling the advancement of genome editing therapies in IRDs

Base editing enables the precise correction of point mutations in genomic DNA through single-nucleotide conversions without inducing DSBs or requiring donor DNA templates (**Figure 1**). Notably, this technology offers superior editing efficiency in quiescent cells (39) and minimizes undesired indel byproducts and DSB-related adverse events (32) compared to traditional CRISPR/Cas nucleases.

Base editors consist of a Cas nickase with one inactive nuclease domain, tethered to a deaminase enzyme, and loaded with a gRNA. Depending on the identity of the deaminase enzyme and other architecture specificities, they can be engineered to correct C•G-to-T•A (cytidine base editors, CBE) (40), A•T-to-G•C (adenine base editors, ABE) (41), or even C•G-to-G•C (CG base editors, CGBE) (42–44) point mutations. When delivered to cells, base editors scan the genomic DNA for a region complementary to the gRNA and containing the appropriate PAM sequence, typically within a ~15 nt window. Upon recognition of the target site, the Cas protein exposes a single-stranded DNA bubble, which is then readily deaminated by the fused deaminase enzyme, inducing targeted single-base conversion. Finally, the active nuclease domain selectively nicks the non-deaminated strand to bias cellular mismatch repair to replace the unedited strand using the edited strand as a template.

The advent of base editing has unlocked new possibilities for the targeted correction of pathogenic point mutations and inspired significant research efforts to bring this technology from bench to bedside, particularly for ocular applications. In 2020, Levy et al. devised a split-intein base editor dual-AAV system that achieved therapeutically relevant editing efficiencies in the mouse retina, at viral dosages well-tolerated in humans (45). After subretinal injection, the optimized split-CBE and split-ABE AAVs yielded average editing efficiencies of 19% C•G-to-T•A and 26% A•T-to-G•C among rod photoreceptors, respectively. However, CBE delivery to retinal cells resulted in a high frequency of indel mutations in non-base edited alleles, reaching up to 34% (45). Later, Jang et al. applied ABE ribonucleoproteins (RNPs) to correct a nonsense point mutation at the *Rpe65* locus in *rd12* mice, an animal model of LCA (46). ABE RNPs were administered to the subretinal space using a commercial non-viral transfection reagent. The average correction efficiencies were just 1.8% in juvenile and 1.2% in adult mice but led to significantly increased levels of *Rpe65* mRNA and restored expression of RPE65 protein. Compared to plasmid-mediated base editing, ABE RNPs generated less bystander editing and fewer off-target effects in both DNA and RNA, which was found to be mainly attributed to the limited lifespan of RNPs in cells (46). Suh et al. have also demonstrated that the subretinal delivery of single lentiviral constructs, co-expressing sgRNA and codon-optimized ABE, into adult *rd12* mice could correct the target mutation with up to 29% efficiency while inducing minimal indel formation and off-target editing (47). Treated mice displayed restored expression of functional RPE65, rescued visual cycle, and near-normal visual and retinal function levels. Moreover, visual cortical responses to various stimulus parameters – including orientation, spatial and temporal frequencies, size, and contrast – were recovered with treatment. In a subsequent study (48), the same ABE lentiviral system was further optimized regarding editing efficiency and specificity, achieving up to 40% functionally rescued alleles with no bystander edits and undetectable off-target activity. Strikingly, this evolved ABE was shown to provide long-lasting protection of cone photoreceptors and counteract vision deterioration in *rd12* mice, even at advanced stages of retinal degeneration deemed beyond the therapeutic window. This finding suggests that base editing could potentially fill the gap of Luxturna supplementation therapy, for which several long-term clinical studies have reported the continuation of retinal degeneration and relapse in visual acuity a few years after treatment (10–12). Most recently, Jo et al. (49) established a dual-AAV system containing split-intein ABEs that secured average editing efficiencies of approximately 6% in the RPE of young *rd12* mice through subretinal injection, with negligible off-target editing in genomic DNA. At six weeks after administration, treated mice showed a significant increase in expression of functional *Rpe65* mRNA (15.2% of that of wild-type mice), restored RPE65 protein expression, and enhanced light-induced electrical responses from retinal tissues (amplitudes of ERG waves were ~60% of those in wild-type mice). Therapeutic effects were sustained even at three months after injection and were comparable to those observed in *rd12* mice receiving AAV-RPE65 gene supplementation. These findings suggest that base editing can provide a therapeutic opportunity in treating LCA.

While base editing has enhanced the potential of CRISPR-based genome editing, it still entails some limitations that significantly constrain its applicability and scope. Concerning targeting range, not all genes are targetable by DNA base editors due to the requirement of a PAM sequence at a specific range downstream of the target nucleotide. Moreover, undesired bystander editing usually occurs when multiple cytidines or adenines lie within the activity window (50). Genome- and transcriptome-wide analyses have also revealed substantial off-target mutations in DNA and RNA (51–53), posing severe implications for using base editors in research and clinical settings. Finally, their target scope is restricted to correcting only 6 out of the 12 possible types of point mutations, excluding insertions, deletions, and most transversions.

## Prime editing reshaping the horizon of inherited retinal disorders

Prime editors are the latest addition to the CRISPR/Cas toolbox (54) and consist of two main components: (1) a Cas nickase fused to a modified reverse transcriptase and (2) a prime editing gRNA (pegRNA) (**Figure 1**). The pegRNA comprises a spacer sequence that directs the Cas complex to the target DNA, a primer-binding site (PBS) to prime reverse transcription, and a reverse transcription template (RTT) encoding the desired modification. In brief, the editing process begins with the binding of the PE/pegRNA complex to the target DNA, followed by the generation of a Cas-induced single-strand break in the PAM-containing strand. Then, the PBS hybridizes to the nicked 3’ end, and the desired edit is introduced by reverse transcription using the RTT as a template. This edited DNA strand is finally incorporated into the genome through endogenous cellular processes that can be promoted by nicking the non-edited DNA strand.

Prime editing is a powerful and highly versatile tool that enables the programmable installation of any single-base substitution, small insertion, or small deletion, potentially addressing up to 89% of human pathogenic genetic variants (54). Like base editors, prime editors operate without inducing DSBs or requiring donor DNA templates, reducing the rate of undesired indels and other DSB-related adverse outcomes, DNA toxicity, and the probability of random integration. Similarly, by relying on cellular mismatch repair mechanisms, prime editors can reverse genetic defects in dividing and non-dividing cells more efficiently than CRISPR-mediated HDR. Remarkably, the requirement of three checkpoint base-pairing events for productive editing grants prime editing unparalleled specificity and negligible off-targeting effects compared to other genome editing technologies. Moreover, as the desired edit is directly copied from the RTT sequence, whose length can be user-defined, prime editors do not cause any bystander edits and are less restricted by the requirement of a PAM sequence near the target site.

As a brand-new technique, prime editing still needs to catch up to CRISPR/Cas nucleases to move into the clinical setting. Nevertheless, emerging preclinical studies show highly precise and efficient correction of disease-causing mutations and compelling evidence of improved visual function and retinal cell survival in animal models of IRDs. For instance, Jang et al. demonstrated that subretinal injection of a dual AAV-split prime editor system (AAV-PE2) in the *rd12* mouse model of LCA could achieve an average editing efficiency of 28% among transduced RPE cells and rescue visual function, without eliciting any detectable unintended edits (55). Most recently, Qin et al. established a dual AAV-split prime editor system with unconstrained PAM requirements (AAV-PE^SpRY^) that achieved over 76% editing efficiency among transduced retinal cells of the *Pde6b* mouse model of RP, with minimal indel formation and off-target activity (56). Treatment with PE^SpRY^ reversed rod and cone photoreceptor cell loss, restored the production of functional PDE6β to near-normal levels, and markedly improved visual function, as substantiated by electroretinogram and behavioral assessments (56).

The development and optimization of prime editors is progressing rapidly, now boasting five generations with ever-increasing efficiency, precision, and target range. Several recent articles review the latest improvements to this system (57, 58), discussing enhanced prime editor effector proteins and pegRNA sequences, as well as strategies to modulate DNA repair mechanisms and improve accessibility of the genomic target site. While this technology is still in its early stages of development, with several challenges yet to be addressed, it offers promising implications for the future of IRD treatment.

## Nanotechnology: a new era for gene therapy

Although genome editing offers unprecedented opportunities to treat IRDs, the lack of efficient and safe delivery systems has greatly hindered successful clinical translation. Until recently, research on ocular gene therapy has mainly focused on AAV vectors, owing to their unparalleled transfection efficiency in retinal tissue and superior safety profile compared to other viral vectors. The early clinical success of Luxturna firmly established AAVs as the leading platform for gene delivery. However, as clinical trials progress, alarming safety concerns arise due to treatment-related serious adverse events in 35% of clinical trials assessing subretinal AAV gene therapies (59). These include persistent intraocular inflammation (5, 6, 12, 60, 61), retinal atrophy (62, 63), and the generation of neutralizing antibodies and cytotoxic T-cells against the vector (12, 64). Besides immunogenicity, another long-standing challenge facing AAVs is their limited cargo capacity (< 5 kb) (65), which hinders the packaging of large transgenes required for CRISPR-based genome editing tools. Although strategies are being developed to overcome this obstacle by splitting transgenes into separate AAV vectors, *in vivo* reconstitution has proven to be highly inefficient thus far. This approach implies co-transduction in the same cell by two independent vectors, recombination in the correct orientation, and expression of a large transgene cassette (66). Therefore, exploring and designing novel classes of nanocarriers has become imperative for advancing the use of emerging editing tools in treating IRDs.

**Figure 2:**
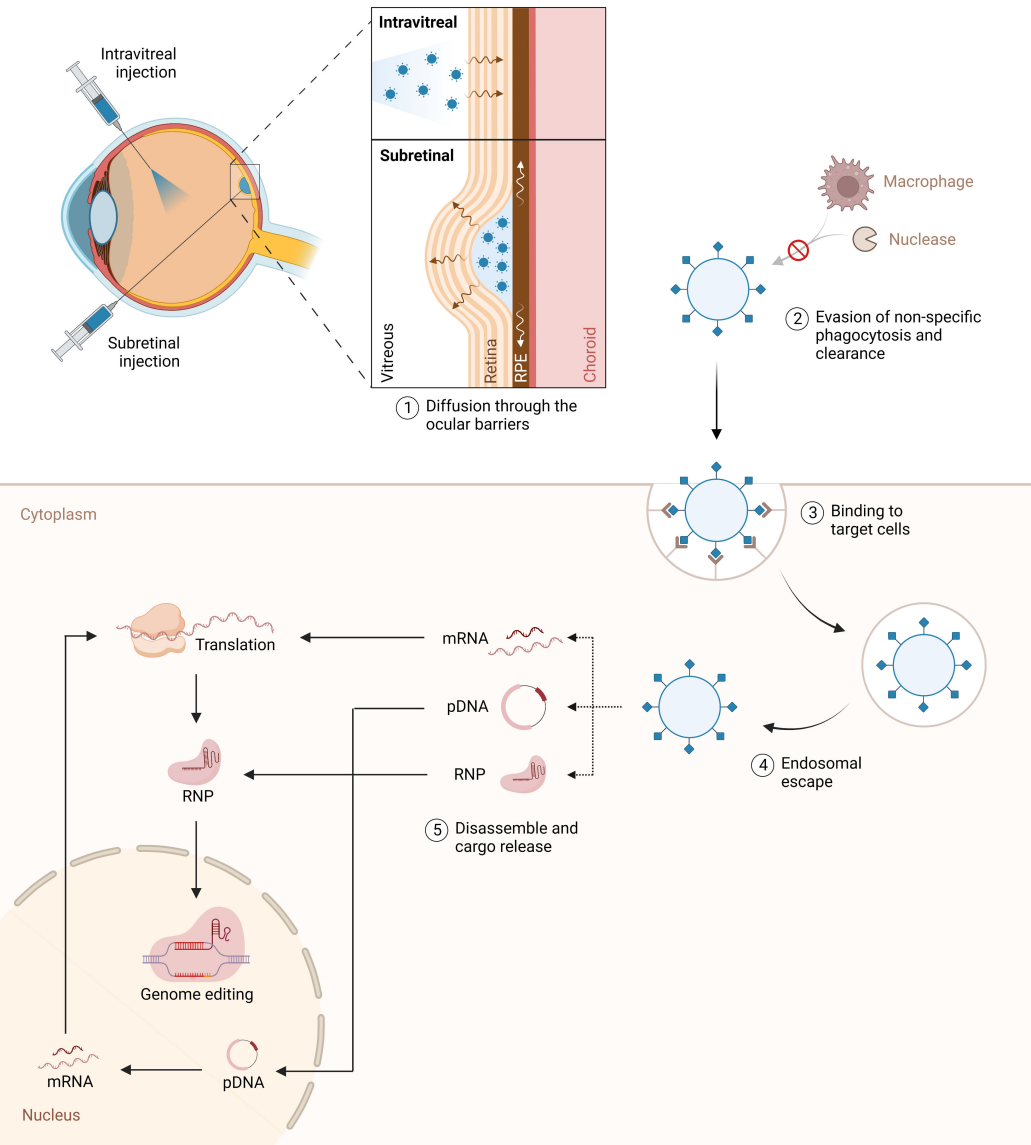
Intravitreal and subretinal nanoparticle delivery for genome editing in the retina. The figure illustrates the targeted delivery of nanoparticles to the retina via intravitreal and subretinal injection techniques. A fundamental challenge is to develop tailored vectors capable of overcoming multiple biological and molecular barriers to efficiently deliver therapeutic agents to target retinal cells in vivo, while minimizing adverse side-effects. The nanoparticles are designed to evade the immune system, enabling efficient and precise transportation of CRISPR-Cas9 cargo to the target cells. Once internalized, nanoparticles must escape endosomes and release their cargo into the cytosol. The nanoparticles carry a diverse payload, including ribonucleoproteins (RNP), mRNA, and DNA, to facilitate genome editing within the retinal cells’ nuclei.

In recent years, nanoparticles have attracted considerable interest as a promising alternative to obviate most of the shortcomings of their viral counterparts (**Table 1**). Notably, these systems offer remarkable versatility, superior packaging capacity, low immunogenicity, long-term stability, and cost-effective manufacturing at large-scale (76). A wide range of nanocarriers are currently being explored in the pursuit of efficient retinal gene delivery, with particular emphasis on lipid-based nanoparticles (LNPs). The fundamental challenge is to develop tailored vectors capable of overcoming multiple biological and molecular barriers to efficiently deliver therapeutic agents to target retinal cells *in vivo*, while minimizing adverse side-effects. Specifically, the vector should: i) cross the physiological barriers of the eye to reach the desired cells; ii) encapsulate and protect its cargo from sequestration or elimination prior to cell entry; iii) facilitate cellular uptake in target cell types; iv) promote endosomal escape, and v) disassemble and release its cargo into the appropriate intracellular compartment (**Figure 2**) (76, 77).

**Table 1:** Summary of state-of-the-art studies on non-viral delivery to retinal cells *in vivo* and *ex vivo*.

Delivery system	Injection	Animal model	Cargo/Approach	Main results	Ref.
Supramolecular nanoparticles conjugated with TAT peptide	Intravitreal	BALB/c mice	pDNA encoding CRISPR/Cas9 components and the *RS1* gene Knock-in of the *RS1* gene	• *RS1* gene was precisely integrated into the desired genome site, resulting in RS1 protein expression	(67)
Carboxylated nanodiamonds covalently bound to linear DNA constructs	Intravitreal	C57BL/6 mice	DNA encoding Cas9, sgRNA and HDR template Knock-in of an XLRS mutation into the *Rs1* gene	• Introduction of the *RS1* mutation into the *Rs1* gene resulted in pathological features typical of XLRS	(68)
pH-responsive SMOF nanoparticles conjugated with ATRA targeting ligand	Subretinal	Ai14 mice	Cas9-sgRNA RNP Knock-out of the Ai14 stop codon	• RNP-loaded SMOF-ATRA nanoparticles enabled efficient genome editing specifically in the RPE	(69)
Engineered VLPs pseudotyped with VSV-G glycoprotein	Subretinal	*rd12* mice	ABE RNP Correction of a nonsense point mutation at the *Rpe65* locus	• Efficient base editing in the RPE with minimal off-target effects, leading to partial recovery of visual function	(70)
3 LNPs with surface charges ranging from neutral to positive (6.2 – 31.2 mV)	Intravitreal	C57BL/6 mice	siRNA *Rbpms* and *Rpe65* gene silencing	• Positive LNPs managed to deliver siRNA to the innermost retinal layers, mediating ~25% gene knockdown in RGC • Neutral and mildly positive LNPs barely delivered siRNA to the retina	(71)
11 LNPs with cationic or ionizable nature and variable degree of saturation of the hydrocarbon tail	Subretinal	BALB/c mice	mRNA Reporter gene transfection	• LNPs with ionizable lipids of low pKa and unsaturated hydrocarbon chains elicited the highest expression • MC3-LNPs were the best-performing	(72)
8 MC3-LNPs that varied in size by changing PEG content	Subretinal Intravitreal	BALB/c mice Ai9 mice *apoE* ^-/-^ mice *Mertk* ^-/-^ mice	mRNA Reporter gene transfection	• MC3-LNPs with lower PEG content and larger size elicited the highest expression levels • Failed to penetrate the ILM and reach the outer retina by intravitreal administration	(73)
MC3-LNPs conjugated with heptameric peptide ligands at varying surface densities	Subretinal Intravitreal	BALB/c mice Ai9 mice Rhesus macaque	mRNA Reporter gene transfection	• Robust expression in the RPE, photoreceptors, and Müller glia by subretinal administration • Failed to penetrate the ILM and reach the outer retina by intravitreal administration	(74)
HA-coated liposomes	Intravitreal	Bovine retinal explant with intact vitreoretinal interface	mRNA Reporter gene transfection	• HA-coating enhanced LNP mobility in the vitreous humor, but failed to overcome the ILM	(75)

ABE, adenine base editor; ATRA, all-trans retinoic acid; HA, hyaluronic acid; HDR, homology-directed repair; ILM, inner limiting membrane; LNP, lipid nanoparticle; LPD, liposome-protamine-DNA complex; NLS, nuclear localization signaling; PEG, polyethylene glycol; RGC, retinal ganglion cells; RNP, ribonucleoprotein; RPE, retinal pigment epithelium; SMOF, silica-metal-organic framework; TAT, cell-penetrating transactivator of transcription peptide; VLP, virus-like particles; VSV-G, vesicular stomatitis virus G envelope glycoprotein; XLRS, X-linked retinoschisis.

### Nanoparticles for delivering genome editing components to the retina

To date, only a limited number of studies have investigated non-viral delivery of CRISPR/Cas systems to the eye. Chou et al. designed supramolecular nanoparticle (SMNP) vectors for codelivery of CRISPR/Cas9 and *RS1* gene, presenting a potential non-viral therapeutic solution for X-linked juvenile retinoschisis (XLRS) (67). The SMNP formulation was optimized through combinatorial screening of different composition ratios and degrees of surface coverage with a membrane penetration ligand (transactivator of transcription peptide, TAT). Results show that intravitreal delivery of the dual-SMNP system into BALB/c mice enabled robust knock-in of full-length *RS1* gene in retinal ganglion cells without negatively affecting retina anatomical integrity (67). In another study, modified nanodiamonds (NDs) were used to deliver CRISPR/Cas9 components and introduce an XLRS-specific mutation in the *Rs1* gene (c.625C>T) (68). The surface of ND particles was functionalized with a carboxyl group and conjugated to a reporter protein via the peptide bond. This reporter protein, in turn, was covalently bound to linear DNA encoding the genome editing agents, thereby enhancing the stability and accessibility of nucleic acids. Before administration, NDs were stabilized by coating with bovine serum albumin to prevent aggregation and improve delivery efficiency. Intravitreal delivery of ND formulation to C57BL/6 mice successfully introduced the desired mutation into the *Rs1* gene, resulting in aberrant photoreceptor structure, a pathological hallmark of XLRS. Although *Rs1* disruption only affected photoreceptor morphology and had no impact on other retinal cell layers exposed to NDs, the authors acknowledge the importance of ensuring specificity towards target cells when designing nanocarriers for genome editing applications. For future use, they propose the replacement of the reporter protein with cell-type specific ligand peptides as a strategy (68). Wang et al. demonstrated the feasibility of this approach by decorating nanoparticles with a targeting ligand, all-trans retinoic acid (ATRA), to deliver Cas9-sgRNA ribonucleoproteins (RNPs) specifically to the RPE (69). They developed a pH-responsive silica-metal-organic framework (SMOF) nanoparticle that enabled pH-controlled release and endosomal escape. ATRA-conjugated SMOF nanoparticles loaded with RNPs mediated robust genome editing *in vivo* in murine RPE via subretinal injection (69).

Recently, viral-like particles (VLPs) have emerged as a compelling candidate for the safe and targeted delivery of genome editing components to the retina (70). These cutting-edge systems exploit viral scaffolds to deliver mRNA, protein, or RNP cargoes instead of viral genetic material, leveraging the efficiency and tissue targeting capacity of viral vehicles while bypassing inherent safety concerns. Banskota et al. developed an engineered VLP (eVLP) platform pseudotyped with vesicular stomatitis virus G envelope glycoprotein for the targeted delivery of therapeutic RNPs to the RPE (70). As a proof-of-concept, eVLPs were loaded with ABE RNPs and applied to correct the nonsense point mutation at the *Rpe65* locus in adult *rd12* mice. A single subretinal injection of ABE-eVLPs supported 12% base editing efficiency with minimal off-target effects, leading to partial recovery of visual function, measured by electroretinography. This performance met or exceeded that of previously reported viral delivery methods, highlighting the therapeutic potential of eVLPs as a powerful and safe tool for efficient genome editing in the retina.

### Optimizing nanoparticles for ocular gene delivery

Non-viral delivery of CRISPR components to the eye holds immense potential for treating ocular diseases through genome editing. Drawing from valuable insights gained from previous studies on non-viral ocular delivery, even those not explicitly aimed at genome editing, can play a pivotal role in shaping future endeavors to develop efficient and targeted vectors for CRISPR-based therapies. Currently, extensive research is dedicated to addressing challenges associated with intravitreal delivery, which offers a less invasive approach compared to conventional subretinal administration. Intravitreal injections, while bypassing the anterior eye barriers and allowing *in situ* delivery, require efficient diffusion through the vitreous and successful traversal of the posterior segment to reach the targeted diseased cell populations (78).

The physicochemical properties of nanoparticles, including size and surface charge, play a crucial role in overcoming eye anatomical barriers (79, 80). Huang and Chau have evaluated three differentially charged LNPs for their ability to deliver siRNA to mice via intravitreal administration (71). Results revealed that the more positive LNPs (31.2 mV) facilitated efficient gene downregulation in retinal ganglion cells, but not in the outermost retinal layers. In contrast, the neutral (6.2 mV) and mildly positive (15.9 mV) LNPs barely delivered siRNA into the mouse retina. This could be the result of fast clearance through the anterior chamber due to insufficient binding to the negatively charged vitreous humor and inner limiting membrane (ILM) (71). Gaurav Sahay lab conducted two more studies that aimed to understand and optimize the critical components of LNPs required for successful penetration to the back of the eye. First, Patel et al. evaluated eleven LNP formulations for their ability to deliver mRNA to the mouse retina *in vivo* via subretinal injection (72). These LNPs differed in their cationic or ionizable nature and degree of saturation of the hydrocarbon tail, both determining factors of mRNA encapsulation efficiency and endosomal escape capacity. Among the assortment of LNPs tested, those containing ionizable lipids of low pKa and unsaturated hydrocarbon chains achieved the highest levels of mRNA expression in the RPE, with the best-performing formulation being an MC3-based LNP. Ryals et al. subsequently extended the work of Patel and colleagues by evaluating eight novel MC3-based LNPs that varied in size by changing polyethylene glycol (PEG) content (73). Overall, intravitreally delivered particles containing less PEG (0.5%), and therefore exhibiting larger size (~150 nm), revealed the highest reporter protein activity. Expression was observed in the optic nerve head, the trabecular meshwork, and, to a lesser extent, in the Müller glia. Importantly, LNPs failed to penetrate the ILM, being sequestered in the vitreous. The signal detected in Müller glia is, therefore, most likely attributable to LNP uptake through the end feet of these cells, which sit at the vitreoretinal interface thus avoiding the ILM.

While the aforementioned studies demonstrate variable degrees of success in overcoming ocular barriers from the humor vitreous, an important shared limitation is the use of rodent models, which are known to have a simplified ILM structure that is underdeveloped compared to that of larger species (81). Herrera-Barrera et al. included a nonhuman primate model, the rhesus macaque, to validate the retina penetration capacity of MC3-based LNPs (74). Building on their previous work (72, 73), they functionalized the surface of MC3-based LNPs with penetrating heptameric peptide ligands that target photoreceptors. Subretinally injected LNPs conjugated with the top-performing peptide mediated robust mRNA expression in RPE, photoreceptors, and Müller glia of rodents and rhesus macaques. However, difficulties persisted in crossing the ILM through intravitreal injection, with these novel LNPs still achieving only very limited expression in Müller glia by this route of administration. Similarly, Devoldere et al. employed bovine retinal explants with intact vitreoretinal interface to evaluate the effect of hyaluronic-acid coating on liposomes’ intravitreal mobility and traversal through the ILM (75). They concluded that although this coating strategy allowed liposomes to successfully surmount the vitreous, it was still not sufficient to ensure retina penetration, as most particles accumulated at the ILM.

## Discussion

The recent surge in research focused on CRISPR-based genome editing marked a paradigm shift in the treatment of IRDs. Genome editing holds the potential to deliver enduring therapeutic effects while encompassing a much wider range of diseases compared to existing gene supplementation therapies. By changing the gRNA sequence, CRISPR-based systems can be rigorously programmed to target virtually any disease-causing mutation, presenting an unprecedented opportunity to address the complex and genetically diverse nature of IRDs. As the field of genome editing continues to advance, a multitude of new therapeutic avenues are being explored in preclinical studies. Nevertheless, before transitioning to clinical trials, these strategies require comprehensive pre-clinical evaluation and must overcome significant challenges related to editing efficiency, safety, and delivery to the eye.

To fully harness the potential of CRISPR-based therapies, engineering enhanced genome editing tools that exhibit superior efficiency and precision is of utmost importance. This pursuit requires the delicate balance between optimizing the efficiency of on-target editing while concomitantly minimizing the incidence of undesired byproducts and off-target mutations. Various strategies have been proposed to refine the editing process, including narrowing the editing window, employing truncated gRNAs, and designing highly specific gRNAs and high-fidelity Cas nucleases. However, achieving a fair and reliable comparison among these approaches requires the establishment of validated pipelines and standardized protocols to monitor efficiency and safety. Also, employing appropriate animal models that closely resemble human physiology becomes imperative for a well-informed assessment of the potential benefits and risks associated with genome editing. *In vitro* conditions offer precise control over the experimental setup – including editing agents’ concentration, exposure duration, and target site accessibility –, facilitating optimal editing conditions and leading to erroneously higher rates of successful gene modification. In contrast, *in vivo* experiments introduce additional challenges. In *in vivo* settings, off-target mutations can occur in unintended cell populations, while delivery vehicles must navigate intricate biological barriers and evade immune system clearance to reach target cells.

Another critical challenge to genome editing lies in developing safe and efficient delivery systems. The application of nanoparticles for treating retinal diseases is still a relatively young and evolving field of research, presenting notable obstacles. The studies discussed in this review (**Table 1**) highlight the inability of current non-viral formulations to deliver gene therapy components to the outer retina through the intravitreal route when tested in models that are more clinically relevant than rodents. Despite optimization attempts, nanoparticles consistently face obstacles in traversing ocular barriers, especially the ILM. The significance of the ILM barrier in diseased retina remains uncertain, as multiple studies suggest potential disruption during retinal degeneration (82–84). Therefore, conducting further therapeutic studies using disease models that faithfully replicate human retinal barriers becomes crucial, enabling a more comprehensive understanding of ILM integrity under pathological conditions and its implications for retinal drug delivery. By combining advancements in genome editing and nanomedicine with consistent guidelines and robust non-clinical models, the field can progress towards the development of CRISPR-based therapies with enhanced outcomes and reduced risks.

While the clinical translation of nanotechnologies is highly anticipated, it is accompanied by several challenges, particularly concerning manufacturing scalability. Many processes and methods currently employed for designing and synthesizing nanoparticles are not suitable for large-scale production (85). This challenge can hinder the transition from laboratory research to commercial products, complicating the ability to meet anticipated clinical demand and maintain consistent product quality. Furthermore, understanding biodistribution and clearance mechanisms is essential to elucidate the complex interplay between nanoparticles and living organisms. On one hand, nanoparticles may elicit toxic or adverse reactions, either directly or due to degradation byproducts. On another, the body’s immune system may promptly recognize and clear nanoparticles, undermining their therapeutic efficacy. Therefore, modifying nanoparticles to evade immune surveillance while retaining their functionality presents a significant hurdle. In light of these multifaceted challenges, it becomes clear that integrating expertise from material science, engineering, and biology will be pivotal to the successful clinical translation of nanoparticle-based interventions.

In conclusion, the advent of novel classes of genome editors has opened highly promising avenues for the treatment of diverse IRDs. To unlock the full potential of genome editing, the development of safe and efficient carriers that can cross multiple retinal barriers is paramount. Nanoparticles offer a safer and more cost-effective alternative with superior packaging capacity compared to AAVs. However, further optimization and improvements are needed to ensure satisfactory transfection efficiency in the retina through less invasive intravitreal route. By synergizing the capabilities of new genome editing tools with the remarkable versatility of nanoparticles, a revolutionary shift awaits the field of IRD therapeutics. This will unequivocally expand the horizons of therapeutic possibilities for IRDs and set the stage for a transformative era of treatments.

## Author contributions

CC: Writing- original draft. LL: Writing- original draft. PA: Conceptualization, Writing- original draft, Writing- review & editing, Supervision. MS: Conceptualization, Writing- review & editing.
